# Parent-Perceived Stress and Its Association With Children’s Weight and Obesity-Related Behaviors

**DOI:** 10.5888/pcd16.180368

**Published:** 2019-03-28

**Authors:** Melanie J. Baskind, Elsie M. Taveras, Monica W. Gerber, Lauren Fiechtner, Chrissy Horan, Mona Sharifi

**Affiliations:** 1 Department of Pediatrics, University of California–San Francisco, San Francisco, California; 2 Harvard Medical School, Boston, Massachusetts; 3 Division of General Academic Pediatrics, Department of Pediatrics, Massachusetts General Hospital for Children, Boston, Massachusetts; 4 Department of Nutrition, Harvard School of Public Health, Boston, Massachusetts; 5 Division of Gastroenterology and Nutrition, Department of Pediatrics, Massachusetts General Hospital, Boston, Massachusetts; 6 Section of General Pediatrics, Department of Pediatrics, Yale School of Medicine, New Haven, Connecticut

## Abstract

**Introduction:**

Psychosocial stress is associated with obesity in adult and pediatric populations, but few studies have examined the relationship between parent-perceived stress and risk of child obesity and related behaviors.

**Methods:**

We studied 689 pairs of parents and children aged 2 to 12 in Massachusetts with a body mass index (BMI) at or above the 85th percentile. Recruitment occurred from June 2014 to March 2015, and data collection ended in March 2016. We asked parents about their perceived stress and categorized responses as low, moderate, or high. We examined associations of parents’ stress with children’s BMI, expressed as a percentage of the 95th percentile (%BMIp95), and obesity-related behaviors by using multivariable regression models adjusted for child and parent characteristics. We stratified results by race/ethnicity, annual household income, and the child’s age.

**Results:**

In fully adjusted models, the association between high versus low parent-reported stress and children’s %BMIp95 remained significant only for children in low-income households (β = 5.12; 95% confidence interval [CI], 0.94–9.30) and for non-Hispanic black children (β = 7.76; 95% CI, 1.85–13.66). Parents with high or moderate stress versus low stress were less likely to report that their children met recommendations for fast-food consumption (high stress, prevalence ratio [PR] = 0.79; 95% CI, 0.65–0.96; moderate stress, PR = 0.70; 95% CI, 0.59–0.82), but parents with high versus low stress were more likely to report meeting daily physical activity recommendations (PR = 1.21; 95% CI, 1.01–1.45).

**Conclusion:**

Among children with overweight or obesity, parent-perceived stress was associated with fast-food consumption and physical activity. Parent-perceived stress was associated with child %BMIp95 among children in low-income households and non-Hispanic black children. Obesity interventions should consider parent-perceived stress and potential differences in the nature of stress experienced by parents of different racial/ethnic and socioeconomic backgrounds.

**Summary**

**What is already known about this topic?**

Although the causes of obesity are multifactorial, stress is known to be associated with obesity in adult and pediatric populations.

**What is added by this report?**

Our study provides more insight into the relationship between parents’ stress and children’s obesity. We found that among non-Hispanic black and low-income families, higher parent-perceived stress was associated with greater BMI among children.

**What are the implications for public health practice?**

The nature and effect of parent-perceived stress on children’s health may be variable among different populations. Further understanding of this variability is needed to guide meaningful screening for psychosocial mediators of childhood obesity and the development of childhood obesity interventions that are responsive to family context.

## Introduction

Psychosocial stress is associated with obesity in adults and children (1–3). However, few studies have examined stress among parents and its association with their children’s risk of obesity (4–6). Behavioral, biological, and developmental mechanisms support such an association. Parents with high stress may exercise less, eat less healthy diets, and have less time to supervise their children, take them to organized physical activities, and prepare meals for them (6–10). Parents’ stress may affect children’s hypothalamic–pituitary–adrenal axis or disrupt the formation of secure attachment and self-regulation skills (11). A better understanding of the relationship between parent stress and child obesity can support the development of more effective screening and interventions.

Most studies examining the health effects of stress measure a person’s exposure to known stressors, such as physical and mental health, home and family life, and finances. Although this objective approach has advantages, it does not consider how personal and contextual factors, such as resiliency and social support, modify how stressful life events are experienced. Subjective measures of perceived stress capture distinct information on the experience of stress, which may be an important mediator of health outcomes (6). Yet, subjective measures are infrequently used, and few studies have examined the relationship between perceived stress and obesity and potential intergenerational effects. Fewer studies have examined single-item measures of perceived stress that could feasibly be used as a screening tool in primary care settings (12).

The objective of our study was to examine the extent to which parents’ responses to a single-question measure of stress was associated with their children’s body mass index (BMI), defined as a percentage of the 95th percentile (%BMIp95), and weight-related behaviors among children with overweight and obesity.

## Methods

### Participants and setting

We conducted a cross-sectional analysis of baseline survey data from 689 parent–child pairs from 6 pediatric primary care practices in Massachusetts participating in the Connect for Health study — a 2-arm randomized controlled trial studying an obesity intervention that leverages clinical and community resources (13). Recruitment occurred from June 2014 to March 2015, and data collection ended in March 2016. Pediatricians received an alert in the electronic health record to refer patients aged 2 to 12.9 with BMI at or above the 85th percentile for age and sex to the study during well-child visits. Children were eligible if at least one parent or guardian had an active email address, was comfortable reading and speaking in English, and was able to follow study procedures for 1 year. Children were excluded if the family planned to leave the practice, a sibling was already enrolled in the study, the child or parent was a member of the study’s advisory group, or the child had a chronic condition or used medications that interfered with growth. Of the 1,752 children referred by providers, 1,485 met inclusion criteria. We analyzed data from 689 parents who completed the study’s baseline survey (Table 1) with complete responses; the participation rate was 46% (689/1,485). All study activities were approved by the Partners Human Research Committee, the institutional review board of Partners HealthCare.

**Table 1:** Survey Questions Completed by Parents of Children Aged 2 to 12 With Overweight or Obesity, in Eastern Massachusetts (N = 689), Connect for Health Trial, 2014–2016

Variable	Survey Question (13)	Variable Type
Screen time	On a typical day how much time does [child’s name] spend watching television or videos?	Categorical (≤2 h or >2 h/d)
	On a typical day, how much time does [child’s name] spend playing games displayed on media, such as television, desktop computers, laptops, portable DVD players, iPads, or smartphones?	
Sleep duration	In the past week, on average, how much time did [child’s name] sleep during a usual 24-hour period?	Categorical (age-specific^a^)
Physical activity	In the past week, on how many days was your child physically active for a total of at least 60 minutes per day?	Categorical (<7 or 7d/wk)
Fruit and vegetable consumption	Yesterday, did your child eat any fruit? (If yes, parent was then asked the number of times.)	Categorical (<5 or ≥5 servings per day)
	Yesterday, did your child eat any vegetables? (If yes, parent was then asked the number of times.)	
Fast food consumption	In the past month, how often did your child eat something from a fast-food restaurant like McDonald’s, Burger King, Taco Bell, Dunkin’ Donuts or a pizza place?	Categorical (<1 or ≥1 time/wk)
Sweet beverage consumption	Yesterday, did your child drink fruit juice? Fruit juice is a drink, which is 100% juice, like orange juice, apple juice, or grape juice. (If yes, parent was then asked the number of times.)	Categorical (0 or ≥1 serving/d)
	Yesterday, did your child drink any punch, Kool-Aid, sports drinks, Goya juice, or other fruit-flavored drinks, not including fruit juice? (If yes, parent was then asked the number of times.)	
	Yesterday, did your child drink any regular (not diet) sodas or soft drinks, including Malta? (If yes, parent was then asked the number of times.)	

a Aged 2y, ≥12 h/d; 3–4 y, ≥11 h/d; 5–12 y/d, ≥10 h/d (15).

### Measures

**Dependent variables**. Dependent variables were %BMIp95, screen time, sleep duration, physical activity, and consumption of fruits and vegetables, fast food, and sweet beverages. Although BMI *z* score and %BMIp95 are comparable measures in normal weight samples, we selected %BMIp95 as our outcome measure because it outperforms BMI *z* in accurately reflecting adiposity among children with high levels of obesity, as in our sample (14). We calculated %BMIp95 by using height and weight measured by medical assistants and entered into the electronic health record during annual well-child visits. We dichotomized all health behaviors by the health behavior goals of the Connect for Health study (13) (≤2 h/d of screen time, ≥60 min/d exercise on 7 d/wk, ≥5 servings/d of fruits and vegetables, <1 time/wk consumption of fast food, zero consumption of sweet beverages). The goal for hours of sleep per night varied by age, as defined by the National Heart, Lung, and Blood Institute (15). 

**Independent variable**. For our main independent variable, we asked parents a single question, adapted from the Growing Up Today Study, about their perceived stress (16). Parents were asked “How much stress do you feel in your life?” with 5 response options: “I never feel stress,” “I sometimes feel a little stress, but it’s no big deal,” “I feel stress fairly often,” “I sometimes feel a lot of stress,” and “I feel a lot of stress most of the time.” This simple stress measure was chosen as a potential screener that could feasibly be used in a primary care setting. For our analysis, we collapsed the 5 response options into 3 categories: “never/sometimes a little” for low stress, “fairly often/sometimes a lot” for moderate stress, and “most of the time” for high stress.

**Covariates**. Multivariable models included child age, sex, and race/ethnicity; annual household income; and parent BMI (4–6,17,18). Child race/ethnicity was categorized as non-Hispanic white, non-Hispanic black, Hispanic/Latino, or multiracial/other. Parent BMI was calculated as parent-reported weight in kilograms divided by height in meters^2^ and dichotomized to less than 30 kg/m^2^ or 30 kg/m^2^ or more. We chose these cutoff points because nearly half (44%) of parents in our sample had obesity. Parent-reported annual household income was dichotomized to at or less than $50,000 or more than $50,000, given that 40% of the median neighborhood household income for this population sample was $70,000 or more.

**Statistical analysis.** We first calculated mean and standard deviation, or frequency and percentage, for child, parent, and household characteristics at baseline. We then examined the extent to which moderate and high parent-perceived stress, compared with low stress (reference group), was associated with our outcomes. Significance was established at *P* < .05. To test the association between parent-perceived stress and age- and sex-adjusted %BMIp95, we used robust regression with bisquare weighting, an alternative to least square regression, which accounts for outlier observations of the population under study. We adjusted for child race/ethnicity, parent BMI, and household income. To test the association between parents’ stress and children’s health behaviors, we used generalized linear models with the log–binomial distribution to calculate adjusted prevalence ratios (PRs) and 95% confidence intervals (CIs) (19). For the health behaviors, we additionally adjusted for child age and sex. All adjusted log-binomial models converged. To examine whether the effect of parent-reported stress on %BMIp95 and the 5 obesity-related behaviors varied according to sociodemographic characteristics, we included interaction terms and developed stratified models for 3 participant characteristics: child race/ethnicity, annual household income, and child age (2 to <7 and ≥7). Interaction terms were considered significant if *P *values were < .10; therefore, up to 6 significant interaction tests would be expected on the basis of chance alone (20,21). All analyses were conducted in SAS version 9.4. (SAS Institute, Inc).

## Results

Of 689 parents, 301 (43.7%) reported low stress, 246 (35.7%) reported moderate stress, and 142 (20.6%) reported high stress (Table 2). The mean BMI percentile was 95.4 (standard deviation [SD] 4.0), and the mean %BMIp95 was 108.8% (SD, 16.5%). Annual household income was $50,000 or less for 44.1% of respondents; 34.4% of children were non-Hispanic white, 33.8% non-Hispanic black, and 22.1% Hispanic/Latino. The proportion of children who met the health behavior goals at baseline ranged from 11.1% for fruit and vegetable consumption to 51.0% for fast food intake.

**Table 2:** Characteristics of Participants (N = 689) Children Aged 2 to 12 With Overweight or Obesity in Eastern Massachusetts, Connect for Health Trial, 2014–2016

Characteristic	Value
**Parent and Household**	
**Relation to child, n (%)**	
Mother	611 (88.7)
Father	66 (9.6)
Other	12 (1.7)
**BMI^a^, n (%)**	
<30	386 (56.0)
≥30	303 (44.0)
**Annual household income, n (%), $**	
≤50,000	304 (44.1)
>50,000	385 (55.9)
**Parent-perceived stress, n (%)**	
Low	301 (43.7)
Moderate	246 (35.7)
High	142 (20.6)
**Child**	
**Age, y, mean (SD)**	8.0 (3.0)
**BMI percentile, mean (SD)**	95.4 (4.0)
**%BMIp95, mean (SD**)	108.8 (16.5)
**Sex, n (%)**	
Male	332 (48.2)
Female	357 (51.8)
**Race/ethnicity, n (%) **	
Non-Hispanic white	237 (34.4)
Non-Hispanic black	233 (33.8)
Hispanic/Latino	152 (22.1)
Multiracial or other	67 (9.7)
**Child Health Behaviors, n (%)**	
Screen time, ≤2 h/d	163 (23.8)
Sleep (age-specific^b^)	222 (32.3)
Physical activity, ≥60 min/d, 7 d/wk	264 (38.7)
Fruit and vegetable consumption, ≥5 servings/d	74 (11.1)
Fast food consumption, <1 serving /wk	351 (51.0)
Sweet beverage consumption, <1 serving/d	259 (38.1)

Abbreviations: %BMIp95, percentage of the 95th percentile; BMI, body mass index; SD, standard deviation.

a Calculated as weight in kg divided by height in meters^2^; mean (SD) BMI was 30.3 (6.9).

b Aged 2y, ≥12 h/d; 3–4 y, ≥11 h/d; 5–12 y, ≥10 h/d (15).

In fully adjusted models, there was no significant difference in child %BMIp95 among parents reporting high versus low stress (β = 1.85; 95% CI, −1.03 to 4.73) or moderate versus low stress (β = 1.16; 95% CI, −1.26 to 3.57). However, non-Hispanic black race/ethnicity (*P* = .09) and low household income (*P* = .03) were significant interactions with parent-perceived stress. In stratified models, parents reporting high versus low stress had children with higher child %BMIp95 in households with an annual income of $50,000 or less (β = 5.12; 95% CI, 0.94–9.30]) and among children of non-Hispanic black race/ethnicity (β = 7.76; 95% CI, 1.85–13.66) but not in households with an annual income of more than $50,000 (β = −2.48; 95% CI, −6.55 to 1.59), nor among children of other racial/ethnic groups. There were no significant associations between moderate versus low parent-reported stress and children’s %BMIp95 in these stratified groups. No significant association was found between parent stress and child %BMIp95 when the data were stratified by child age (Figure).

**Figure:**
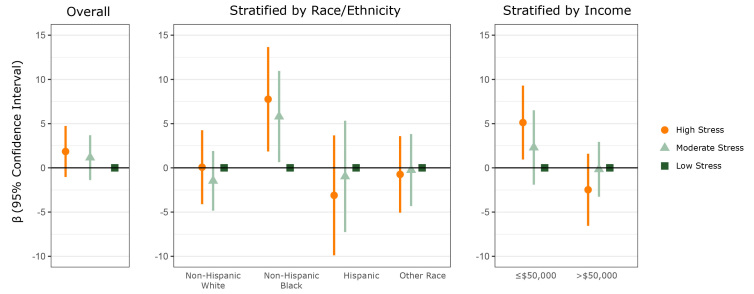
Association between child’s age and sex-adjusted body mass index (BMI), calculated as percentage of 95th percentile (%BMIp95), and parent’s moderate or high stress, compared with low stress, overall and stratified by race/ethnicity and income. Model is adjusted for the child’s race/ethnicity, annual household income, and parent’s BMI. Vertical lines transecting circles, triangles, and squares indicate confidence intervals. Confidence intervals that do not cross zero indicate significance.

**Table:** 

Parent Stress Level	Child BMI at or Above the 95th Percentile, β (95% Confidence Interval)
**Overall**	
High	1.85 (−1.03 to 4.73)
Moderate	1.16 (−1.26 to 3.57)
Low	1 [Reference]
**Annual Household Income, $**	
**<50,000**	
High stress	5.12 (0.94 to 9.30)
Moderate stress	2.3 (−1.78 to 6.39)
Low stress	1 [Reference]
**≥$50,000**	
High stress	−2.48 (−6.55 to 1.59)
Moderate stress	−0.16 (−3.14 to 2.81)
Low stress	1 [Reference]
**Child Race/Ethnicity**	
**Non-Hispanic white**	
High stress	0.07 (−4.11 to 4.25)
Moderate stress	−1.47 (−4.72 to 1.78)
Low stress	1 [Reference]
**Non-Hispanic black**	
High stress	7.76 (1.85 to 13.66)
Moderate stress	5.8 (0.77 to 10.83)
Low stress	1 [Reference]
**Hispanic**	
High stress	−3.11 (−9.89 to 3.67)
Moderate stress	−0.97 (−7.13 to 5.20)
Low stress	1 [Reference]
**Multiracial/other race**	
High stress	−0.74 (−5.07 to 3.58)
Moderate stress	−0.25 (−4.20 to 3.69)
Low stress	1 [Reference]

Parents were less likely to report that their children consumed fast food less often than once per week if parent-perceived stress was high (PR = 0.79; 95% CI, 0.65–0.96) or moderate (PR = 0.70 ; 95% CI, 0.59–0.82) versus low (Table 3). High versus low parent stress was associated with a higher likelihood of parents reporting that their children were physically active for 60 or more minutes daily, 7 days per week (PR = 1.21; 95% CI, 1.01–1.45), but moderate versus low parent stress was not (PR = 0.90; 95% CI, 0.75–1.08). We observed no significant relationship between parent-reported stress and the likelihood of children meeting health goals for screen time, sleep duration, and consumption of fruits/vegetables and sweet beverages (Table 3).

**Table 3:** Multivariable Models of Parent-Perceived Stress and Child Body Mass Index^a^ and Obesity-Related Behaviors Among Children Aged 2 to 12 (N = 689) With Overweight or Obesity, Connect for Health Trial, Eastern Massachusetts, 2014–2016

Variable	Low Stress	Moderate Stress	High Stress
BMI, β (95% CI) [*P* Value^b^]	1 [Reference]	1.16 (−1.26 to 3.57) [.35]	1.85 (−1.03 to 4.73) [.21]
Screen time, ≤2 h/d, PR (95% CI) [*P* value^c^]	1 [Reference]	1.11 (0.84 to 1.48) [.46]	1.04 (0.72 to 1.50) [.85]
Sleep (age-specific^d^), PR (95% CI) [*P* value^c^]	1 [Reference]	0.80 (0.63 to 1.01) [.06]	0.84 (0.64 to 1.11) [.22]
Physical activity, ≥60 min/d, 7 d/wk, PR (95% CI) [*P* value^c^]	1 [Reference]	0.90 (0.75 to 1.08) [.27]	1.21 (1.01 to 1.45) [.04]
Fruit and vegetable consumption, ≥5/d, PR (95% CI) [*P* value^c^]	1 [Reference]	1.30 (0.81 to 2.08) [.28]	0.80 (0.41 to 1.56) [.51]
Fast food consumption, <1/wk, PR (95% CI) [*P* value^c^]	1 [Reference]	0.70 (0.59 to 0.82) [<.001]	0.79 (0.65 to 0.96) [.02]
Sweet beverage consumption, <1/d, PR (95% CI) [*P* value^c^]	1 [Reference]	1.01 (0.82 to 1.24) [.94]	1.05 (0.81 to 1.36) [.72]

Abbreviations: BMI, body mass index; PR, prevalence ratio.

a The percentage of age- and sex-adjusted body mass index at the 95th percentile.

b *P* value determined by robust linear regression with bisquare weighting adjusted for child age, sex, race/ethnicity, annual household income, and parent BMI (weight in kg divided by height in meters^2^; α = .05.

c *P* value determined by multivariable logistic regression adjusted for child age, sex, race/ethnicity, annual household income, and parent BMI; α = 0.05.

d Aged 2y, ≥12 h/d; 3–4 y, ≥11 h/d; 5–12 y, ≥10 h/d (15).

We found a significant interaction between moderate versus low parent stress and Hispanic race/ethnicity in our model of fast food consumption (*P* = .06) and for high versus low parent stress and the child’s age (2–6 vs 7–12) in our model of sweet beverage consumption (*P* = .02). However, stratified models did not show any clear differences between these groups in the effect of parents’ stress on obesity-related behaviors.

## Discussion

In our sample of children with overweight and obesity, we observed, after adjusting for potential confounders, that high versus low parent-reported stress was associated with child %BMIp95 only among children in low-income households and among non-Hispanic black children. Of the 6 obesity-related health behaviors studied, only fast food consumption and physical activity were significantly associated with high versus low levels of parent-perceived stress.

To our knowledge, our study is the first to examine and observe effect modification of the relationship between parent-perceived stress and child BMI by non-Hispanic black race/ethnicity and by household income, a finding with potential implications for psychosocial stress screening and obesity interventions. A prior study observed significant interaction effects of Hispanic race/ethnicity on the association between parent-perceived stress and children’s BMI (5). We did not observe this interaction, which could be a result of our smaller sample size or eligibility requirements, or it could indicate that relationships between parent stress and child BMI are context dependent.

Our results suggest that nonspecific measures of perceived stress may be inadequate for routine screening. The drivers of the heterogeneity of the effect we observed among certain subgroups in our sample are unclear. We found no evidence of effect modification by race/ethnicity or household income of the relationship between parent stress and the studied health behaviors, though these results were limited by smaller sample sizes and underpowered to detect clinically meaningful differences. Unmeasured confounders of the observed association between parent’s stress and children’s BMI may include mental health status, access to community resources, and proximity to green spaces and grocery stores.

A person’s perceived stress may have variable sources and consequences, indicating a limitation of a nonspecific, single-item screener. For example, discriminatory stress, the notion that the experience of prejudice or discrimination is an allostatic load that sets into motion a series of physiological responses (22), may have disproportionately affected both the low-income families and non-Hispanic black families in our sample. The effect of stigma-associated stress on health outcomes, described in the literature as minority stress, warrants more attention in health care research and interventions (22,23). Perceived discrimination is associated with higher rates of obesity (24), and perceived racism has been separately linked to obesity and hypertension (25,26). High stress in groups that experience discrimination, consciously or unconsciously, may be associated with higher levels of obesity through biological and behavioral mechanisms. Discriminatory stress may have a greater effect on the hypothalamic–pituitary–adrenal axis than other types of stress, and the emotional effect may affect health behaviors.

Our findings corroborate and expand on — through identification of effect modification — a prior cross-sectional study among children with and without elevated BMI that used a different single-item measure of perceived stress and similarly observed no association between parent-perceived stress and child obesity in fully adjusted models (6). Another study that studied associations between the 4-item Perceived Stress Scale and child BMI used both cross-sectional and prospective data over a 4-year period and found that the association between parent-perceived stress and child BMI remained significant in both analyses after adjusting for age, sex, race/ethnicity, and community of residence but was substantially attenuated after adjusting for additional factors (5). Together, these studies suggest that the relationship between parent-perceived stress and child obesity is confounded by child, parent, community, and household characteristics.

Our finding that children of parents with high levels of stress were more likely to consume fast food once a week or more is consistent with our hypothesis and the findings of prior research (6). Stress is associated with higher consumption of high-fat and high-sugar foods and may be associated with less time to prepare food at home, which may drive food-purchasing choices (10,27). As a modifiable risk factor of obesity, pragmatic strategies to reduce fast food consumption should consider parent stress.

In our sample, the observation that parents with high versus low stress were more likely to report daily physical activity in their children was an unexpected finding that has not previously been observed (6). In fact, a recent systemic review found consistent evidence for associations between high maternal stress and low child physical activity (28). The results of our study may be specific to how the survey question was asked. Parents with more stress may have less face-to-face time with their children and overestimate their level of physical activity. If our results represent a true association, this could be due to the children’s involvement in organized activities, leading to greater parent stress secondary to associated costs, time, and organization required for participation. Future studies may benefit from objective measures of physical activity, such as accelerometry.

We found no significant associations between parent stress and other behaviors, and to our knowledge this is the first study to look at the association between parent-perceived stress and child screen time, sleep duration, and consumption of sweet beverages; we found no significant associations between parent stress and these other behaviors. A qualitative study found that parents may use screen time with their kids as a way of decreasing their own stress (10), and thus we hypothesized an association between high parent stress and child screen time; we found no such association.

Beyond the inclusion of multiple obesity-related behaviors, a strength of this study is the use of %BMIp95, as is newly recommended for use in studying populations with severe obesity, rather than BMI z score (14). An additional strength is the use of a single question to measure perceived stress, which, unlike objective measures of cortisol levels or long surveys, may feasibly be asked as a part of routine well-child visits (16). Recently, an Institute of Medicine committee evaluated several stress measures in the literature for inclusion in the electronic health record as part of a new set of psychosocial vital signs. They recommended a single-item measure rather than a long screening tool, based on greater usefulness and feasibility (12). Our results suggest that a nonspecific, single-item screener for stress should not stand alone but could be part of a multistep screener, wherein a positive single-item screen triggers follow-up questions and discussions to determine sources of stress and potential effect on child health behavior and outcomes.

Our study had limitations. Its cross-sectional design limited conclusions about directionality. We lacked information on the validity and reliability of the measure of stress used in this study, yet our observed associations with children’s BMI and fast food intake are consistent with those of prior studies using longer, validated stress measures, which suggests criterion validity. Our analysis of effect modification using interaction terms was constrained by our sample size, particularly for health behaviors. With more testing, we increased the risk of discovering significant results attributable to chance alone. To address this, we formed a priori hypotheses and concurrently conducted stratified analyses to evaluate effect modification. Our sample did not include uninsured children — a relevant population with likely high levels of stress — which limits the generalizability of our results to insured children with overweight and obesity seen in primary care. Data on household size, marital status (4,17,18), education, parents’ mental health, and employment status were not available for our sample and may affect the observed relationships. There was potential selection or response bias because parents with high stress may have been less frequently referred to our study by providers, more difficult to reach, or less willing to participate. Apart from child height and weight, all data were reported by parents, introducing potential reporter bias. When asked about behaviors, parents may have provided socially desirable responses or may not have counted items not explicitly provided as examples (eg, not including flavored milk as a sweet beverage).

In conclusion, we found that parents with high versus low perceived stress were more likely to report more child fast-food consumption and physical activity, and parent-perceived stress was variably associated with children’s BMI depending on race/ethnicity and household income. Further understanding of the drivers of the heterogeneity in the effect of parent-perceived stress on children’s BMI, particularly among children from low income households and children of non-Hispanic black race, is needed to guide meaningful screening for psychosocial mediators of childhood obesity and the development of childhood obesity interventions that are responsive to family context. A single-item measure of stress may be an effective place to start, yet our results suggest a need for further questioning for those parents who screen positive for perceived stress.
